# Radioactivity Measurements on Glazed Ceramic Surfaces

**DOI:** 10.6028/jres.105.031

**Published:** 2000-04-01

**Authors:** Thomas G. Hobbs

**Affiliations:** National Institute of Standards and Technology, Gaithersburg, MD 20899-3541

**Keywords:** alpha, ceramics, electret ion chamber, glazes, radioactivity, scintillation counter, thorium, uranium

## Abstract

A variety of commonly available household and industrial ceramic items and some specialty glass materials were assayed by alpha pulse counting and ion chamber voltage measurements for radioactivity concentrations. Identification of radionuclides in some of the items was performed by gamma spectroscopy. The samples included tableware, construction tiles and decorative tiles, figurines, and other products with a clay based composition. The concentrations of radioactivity ranged from near background to about four orders of magnitude higher. Almost every nuclide identification test demonstrated some radioactivity content from one or more of the naturally occurring radionuclide series of thorium or uranium. The glazes seemed to contribute most of the activity, although a sample of unglazed pottery greenware showed some activity. Samples of glazing paints and samples of deliberately doped glass from the World War II era were included in the test, as was a section of foam filled poster board. A glass disc with known ^232^Th radioactivity concentration was cast for use as a calibration source. The results from the two assay methods are compared, and a projection of sensitivity from larger electret ion chamber devices is presented.

## 1. Introduction

The need for a large-area, low-intensity alpha test source for large face area ion chambers triggered memories: first, site and area measurements with sensitive radiation monitors at the National Institute of Standards and Technology (NIST) and its predecessor agency, the National Bureau of Standards (NBS) had indicated that the general radiation level was higher in an area lined with glazed ceramic tile than in areas lined with wallboard, cinder block, or other construction materials; and, second, that some dinnerware, e.g., pre-World War II Fiestaware, contained significant levels of radioactivity in materials used as coloring agents. A test of alpha emissions from samples of glazed tiles used in construction at NIST/NBS suggested that an examination of emissions from commercially available floor and wall tiles might yield useable information for alpha check sources. The extension of the test to samples of older Fiesta dinnerware led to an investigation of emissions from a variety of pottery and similar items.

For over a century and a half, soils containing naturally occurring radioactive materials have been used in manufacturing glass and ceramic products [1], as base material, as coloring agents, and for other properties, even before the contained radioactivity was identified as such. Artificial teeth, jewelry—especially cloisonné jewelry, wall tiles, electrical materials, and other manufactured articles have also had radioactive materials incorporated into them for various reasons [2]. Recognition of the hazards inherent in such applications led to regulations governing incorporation of byproduct radioactive materials, i.e., materials made radioactive through regulated activities [3], and of source materials, i.e., uranium and thorium [4], into products.

## 2. Materials Tested

As this project evolved, the variety of materials involved led to confusion in describing items and made it obvious that some understanding of nomenclature for ceramic items was necessary. Various sources [5,6,7,8] were used to prepare a list of ceramic descriptions and definitions for adequate presentation of identifying information for the items examined for radioactivity concentrations in this test. These identifiers, in some cases combinations of definitions or descriptions found in the references, are shown in Table 1.

Samples of dishes and other table items, decorative figurines, greenware, floor tiles, wall tiles, and glass samples were begged, borrowed, bought, or brought from the author’s home for analysis. Identification of the radionuclides for certain items with sufficient photon activity was performed on an intrinsic germanium gamma spectroscopy system^1^,^2^. The gamma spectroscopy tests showed those identified to be primarily from the natural radioactive series of ^232^Th and ^238^U. Two samples demonstrated emissions from ^235^U. Almost every sample of glazed and unglazed ceramic items examined exhibited measurable emissions of alpha radioactivity above background, and the range of activities was remarkable; a variation of about four orders of magnitude in levels was observed, more or less depending on the detection method. No analysis gave any evidence that the series progeny were not in equilibrium.

Other than the tile samples, few of the samples had a completely flat surface so that the detectors used could be placed on the sample with a geometry that was reproducible from sample to sample. Thus, the data shown, especially the uncertainties, cannot be called absolutely evaluated for activity concentrations. The uncertainties shown as standard deviations are calculated using only the data from the measurement results, including the background uncertainties; no other sources of uncertainties are included. However, the results definitely indicate a wide range of activity concentrations and provide some information on whether or not moderate or substantial activity concentrations exist.

Table 2 shows the list of items assayed, categorized into four types of samples, with the identifier terms used for subsequent data presentation and a more complete description with markings on a particular item, if any, indicated in quotation marks. For those items with geometries other than flat, the shape that prevents a full detector-to-surface contact is noted, e.g., rimmed saucer indicates that the dish had a raised edge that held either or both of the detector faces up from the saucer’s interior flat surface. For those samples measured with the gamma spectrometry system, the nuclide series generating the activity observed is noted. For most of the samples measured, characteristics of the ^232^Th and the ^238^U families were seen. The green Fiesta tray and the Limoges sugar bowl showed, in addition to ^232^Th series activities, a ^235^U component.

To examine radioactivity distributions throughout the pottery material, measurements were made on the back side of some of the tile samples, to be compared with the measurement result on the front, or glazed, face side. Also, for some samples, a sheet of ordinary copying paper was inserted between the detector face and the item’s surface, to demonstrate that the primary measured radioactive emissions were alpha particles.

There were a number of interesting sidebars associated with the samples accumulated for this project. One plate had been on the bottom of the Delaware Bay for almost a century and a half. It was retrieved by a scuba diver from the wreckage of a sunken ship. The company named on the plate’s reverse, J. W. Pankhurst, had sent three shiploads of china from England to America in the mid-19th century. All three ships had sunk in the bay in a fierce storm; the company went out of business from that loss. Three pieces of glass were from World War II experiments at the National Bureau of Standards on doping glass with rare earths. The glass slabs had been discarded after testing and the work space assigned to another agency. One of the new occupants retrieved the slabs from a scrap pile and used them for bookends for several decades. They were accidentally discovered to contain radioactivity through an incidental scan with a personal radiation survey meter. Another serendipitous discovery came from trying to measure background levels on a section of foam filled poster board with a shiny (glazed) surface. The result was so much higher than anticipated that further measurements were performed and the surface, perhaps a clay-based facing material, was determined to contain radioactive materials, albeit at a very low level. A piece of unglazed, unfired green-ware donated by a local pottery craft shop was measured and then, along with some of the other items, was placed in a dishwasher for cleaning—the greenware piece completely disintegrated; fortunately, it did not substantially affect the dishwasher drain lines. A midnight cat scramble resulted in a shattered decorative urn; this led to the contribution of a shard of the remnants.

## 3. Measurement Methods

Alpha pulse counting was performed with a ZnS scintillation device^3^ (ZnS), known to have minimal observable response to beta or photon radiations, at least at normally encountered levels. The circular detector face has a diameter of about 10 cm; a supporting grid over the sensitive area shields a small fraction of the face area from seeing alpha particles. A mask with a 5 cm diameter hole in the center covered the detector face for all measurements in this test. This provided a mechanism to deal more easily with those samples of smaller diameter than the detector’s full face diameter, and avoided any necessity to treat data with any effect from the full webbed-face dimensions. An Am-241 point source with 9050 nuclear transformations per second activity centered on the face showed the probe had about a 35 % counting efficiency for that source in that configuration.

Ion chamber measurements were performed with a windowless electret device^4^ (EIC). Since the large area detector for which the large-area test source was being sought was too large for most of the samples of ceramic items collected for this test, a smaller EIC was devised. Several of the commercially available 210 mL “S” type screw-top radon monitor devices were separated at the midline and the electret-mounting section of each used as a cylindrical ion chamber with a short-term electret at the end of the chamber, facing the source with no intervening window or absorber. The face diameter was about 8 cm and the volume was about 125 mL. The voltage induced by the charge on the electret was the measurement indication. The measured voltage drop was due to ionization from the sample’s beta particles and photons and from environmental radiations, as well as from the alpha particles emitted from the sample.

The smaller ion chamber used for these measurements naturally had less sensitivity than did the larger chambers for which a check source was originally sought. Two prototype large area ion chambers were available; one, a slope-sided device with both ends flattened, one of the faces with the electret fitting and the other face open, had an open-face diameter of about 7.5 cm and a volume of about 550 mL, and the second, with a hemispherical shape truncated by the electret and its fitting, had a face diameter of about 7.5 cm and a volume of about 600 mL. Measurements with these two larger ion chambers, using some of the larger tile samples, indicated that responses were enhanced over the small chamber by factors of about 3 and 3.5, respectively.

## 4. Measurement Data

### 4.1 Concentration Calibration Sample

A glass “hockey puck” with a 0.05 % mass fraction of ^232^Th was cast by an associate of the NIST Surface and Microanalysis Division for use as a calibration source. This is considerably less contained activity than the amount specified in the cited regulations for source material in glazed ceramic tableware. The activity concentration value of 1.78 Bq/g of glass was used to develop response factors in order to convert measurement data from the two different techniques to comparable units. The diameter of the disk was about 10 cm, fitting quite well the requirements for the masked scintillator and the smaller ion chamber faces. For confirming the comparative sensitivities of the small ion chamber and each larger ion chamber as determined with large area tiles, the face of each larger ion chamber, in turn, was centered over the disk with spacers to hold the disk surface just at the chamber face opening.

### 4.2 Measurement Systems’ Properties

#### 4.2.1 EIC Characteristics

Repeated measurement results with the EIC on the cast glass source with known radioactivity content, and tested with repeated measurements on some of the more active unknowns, were shown by regression analysis to result in a calibration factor of

F=[a+bln(MPV)](V/h)(Bq/g),

where *a* = −0.1796, *b* = 0.3832, and *MPV* is the numerical value of the mid-point voltage calculated between the initial voltage *V*_i_ of the measurement, and the final voltage *V*_f_ when both *V*_i_ and *V*_f_ are expressed in volts. The value of a sample’s activity concentration (in Bq/g) for a voltage drop occurring in a time interval *t* expressed in hours is given by

$$C=[(V_{i}-V_{f})t^{-1}]F^{-1}.$$

A nominal value for the background rate for the EIC was about 0.044 V/h with no difference due to voltage as the initial voltage changed from the maximum of about 650 V to the minimum used for this test, about 200 V. If a value of 450 V is chosen as a representative *MPV*, this background is equivalent to an activity concentration of about 0.02 Bq/g.

#### 4.2.2 ZnS Characteristics

Intermittent retrieval of either or both the electronic package or the scintillation probe for use in the radiation safety program forced reestablishment of operating characteristics, i.e., high voltage and discrimination threshold, with the analog dial adjustments, following the return from field use. To expedite the measurements, exact duplications of the previous settings were not pursued, so the background and the response to the calibration source varied from one measurement set to another. Backgrounds and calibration factors were determined for each uninterrupted set of measurements and applied to the sample measurements conducted in that set. Values for background and calibration factors ranged from about 38 counts per hour and 95 counts per hour per Bq/g to 60 counts per hour and 100 counts per hour per Bq/g.

### 4.3 Data Analysis

A number of factors were involved in the treatment of the data. The nonuniform measurement intervals, the interruptions for field use of the ZnS detector system, the variations in background within an uninterrupted measurement set, the discrete radioactivity counts from the scintillator versus the non-discrete nature of the EIC voltage induced by a charge, all introduced unusual characteristics into the data. Attempts to develop statistical characteristics employing the common methods, e.g., from Youden [9], Natrella [10] and Currie [11], exhibited difficulties from the start.

Drs. K. Eberhardt and L. Currie of NIST graciously consented to review the data and provide comments on possible treatments [12,13]. Currie went so far as to examine the heteroscedasticity of some of the background data, i.e., varying standard deviations and statistical weights, and the resulting incompatibilities with ordinary least-squares treatment, and proceeded to deal with measurement limit determinations under these less-than-ideal circumstances.

#### 4.3.1 EIC Data

The variance of any EIC value must be estimated from replication, since the measurement does not represent a collection of discrete countable events. It is quite possible that there is a measurement duration dependence for the variance, leading to the necessity for using weighted least squares in calculations for measurement limits. Assuming a simplistic inverse variation of the variance of the estimated background rate with time, a typical background rate is 0.044 V/h (equivalent to 0.020 Bq/g, using an *MPV* value of 450 V) with a standard uncertainty, i.e., estimated standard deviation, of 0.0109 V/h (equivalent to 0.005 Bq/g). A Type A evaluation of standard uncertainty for a sample’s reported result is the root-sum-of-squares (RSS) combination of the standard deviation from a series of observations on that sample and the standard deviation from the evaluation of the background.

Assuming that a measurement interval of about a week, i.e., 168 h, was chosen for all measurements, an estimate of one of the desired measurement limits could be determined from a weighted subset of background measurements taken in a single measurement interval. The detection decision limit, or Critical Level, *L*_C_, is estimated as 0.020 V/h (equivalent to 0.009 Bq/g) with 95 % confidence. Calculations leading to minimum detectable value, or Detection Limit, *L*_D_, and minimum quantifiable value, or Quantification Limit, *L*_Q_, assume homoscedasticity, so these values cannot be reliably estimated for this data. However, it is estimated that the two values are approximately twice and five times the Critical Level, respectively, or on the order of 0.04 V/h (equivalent to 0.02 Bq/g), and 0.1 V/h (equivalent to 0.05 Bq/g).

#### 4.3.2 ZnS Data

Noting that the measurement data were divided into sets by interruptions when the instruments were removed from the test, a subset of data from a reasonable set of background measurements was used to develop example values for the measurement limits. It should be remarked that the Poisson process is to be employed, with some caveats. For example, an added variance beyond that resulting from the random nature of radioactivity cannot be ruled out; also, the background cannot be assumed to be “well known.”

For the case of a pure Poisson data set, the background rate from the data subset is 38.56 counts per hour (equivalent to 0.4 Bq/g) with a standard uncertainty, i.e., estimated standard deviation, of 0.206 counts per hour (equivalent to 0.002 Bq/g). An iterative weighted least squares analysis and an additive model yield an estimate for an added variance of 0.22 counts per hour with bounds of 0 counts per hour and 0.6 counts per hour, giving a best estimate for background and standard uncertainty of 38.56 ± 0.23 counts per hour. For a given sample, a Type A evaluation of standard uncertainty for a sample’s reported result is the root-sum-of-squares (RSS) combination of the standard deviation from the sample observation, assuming Poisson statistics, and the standard deviation from the evaluation of the background.

Again choosing a measurement interval of about a week for all measurements and using paired measurements of background and sample, an estimate of *L*_C_ is 1.115 counts per hour (equivalent to 0.002 Bq/g) with 95 % confidence. Likewise, *L*_D_ and *L*_Q_ are found to be 2.245 counts per hour and 7.080 counts per hour (equivalent to 0.005 Bq/g and 0.017 Bq/g), respectively.

## 5. Measurement Results

In the tables of measurement data and calculated values that follow, three decimal places are used for comparative purposes, although the information does not warrant that implied accuracy. For most of the samples, the EIC results exceeded the ZnS results. This was expected since the EIC is sensitive to radiations other than alpha particles, while the ZnS has minimal sensitivity to other radiations, and the detectors would be exposed to a variety of radiations from the samples and to the ambient background radiations. For the samples showing very low levels of contained activity, the measurement time was extended to assure that the measurement did, indeed, indicate a positive level of activity concentration, although probably with reduced reliability. The identifiers shown are as given in Table 2. None of the results for the ceramic items indicated a level of radioactivity above that cited in the regulations for source material incorporated into glazed ceramic tableware. The highest measured value, for NBS glass medium, is about half the limit prescribed for glazed ceramic tableware or about equal to the concentration permitted for glassware excluding construction materials.

Table 3a shows the results of the measurements and uncertainty calculations on those items with geometries that permitted adequate information for the calculations. Table 3b shows the data for those items contained in standing walls, so the ZnS system could not be conveniently employed. No construction material samples were available for measurement. However, the EIC data did confirm the memory of higher than normal radiation levels in glazed tile and brick faced areas. Table 3c shows data for those samples that had poor geometries that were not suitable for measurements adequate for uncertainty determinations. The orange Fiesta data illustrate this effect. The measurements were performed on the bottom of the saucer, since the cup has been glued to the saucer as a training and demonstration source of radioactivity. The scintillator rested on the unglazed rim of the saucer bottom, with the opening seeing the surface of the saucer bottom at a distance, while the ion chamber, with a diameter larger than the rim, was positioned so that the rim and a portion of the saucer bottom intruded into the ion chamber volume. The reduced volume for ion chamber measurement could have reduced its sensitivity, while the proximity could have enhanced the response, so comparative analysis between EIC and ZnS data is not possible.

The results of measurements on the backsides of three tiles showed substantially less activity than did the results for the front sides, demonstrating that whatever glazing material is used as a surface coat has a higher radioactivity content than the tile body material. A sheet of paper inserted between the surface of several samples and the detector face indicated that, indeed, most of the response was to alpha particles; only one measurement on paper was made with the scintillator, and that result confirmed the conclusion.

Note that there are no results for the scintillator for measurements of the NIST foyer brick nor the NIST stair tile, for either the regular face measurement or for the paper shielded measurement. This is because of the difficulty involved in mounting the scintillation detector on a vertical surface, as opposed to the relative ease of fixing the ion chamber to the wall. However, the similarity of results between the two methods for the other samples leads to the belief that a scintillation result would also be similar to the ion chamber result for these two surfaces.

The data show the wide range of activity concentrations observed, about four orders of magnitude range for the dish items and nearly as wide for the figures and for the glass samples. The brick and tile samples did not show this magnitude of concentration ranges but the variation was substantial, nonetheless.

In reviewing the data in the tables, recall the caveat that the implied accuracy of the three decimal places is not truly warranted. The expansion is simply to permit adequate comparisons of data elements within the set of measurement results.

## 6. Conclusion

A number of samples of ceramic items commonly found in everyday living were examined for radioactivity content with two different detection and measurement methods. Every one of the examined items was found to have measurable radioactivity, although the levels for some of the items were so near the detector background that the attribute should not be positively assigned. None of the measurements indicated levels in excess of regulated limits for contained radioactivity. Reasonable agreement was observed for results from the two methods, electret ion chamber and ZnS scintillation. As expected, the samples of older Fiesta tableware and construction tiles for vertical surfaces showed substantial concentrations of activity. Samples of doped glass from work at NBS also showed significant levels. Larger EIC devices, for which the activated sample search was initiated, were characterized by using some of the larger flat samples involved in the test.

## Figures and Tables

**Table 1:** Definitions or descriptions of ceramic terms

Term	Definition or description
Brick	Building or paving unit of moist clay hardened by heat
Ceramic	Relating to manufacture of or product of nonmetallic mineral, first shaped, then hardened by heat
China	Vitreous (relating to glass) porcelain ware
Clay	Fine particles of hydrous aluminum silicates and other minerals
Cloisonné	Style of decoration of fired enamel in raised cells, usually on a metal backing
Glaze	Oxide mixture, e.g., alumina and silica, applied to surface of ceramic wares to form a moisture-impervious barrier
Greenware	Unglazed and unfired pottery item
Porcelain	A hard, fine-grained, sonorous (producing sound when struck), nonporous, and usually translucent and white ceramic ware that consists essentially of kaolin (fine white clay, see: Kaopectate), quartz (silicon dioxide), and feldspar (crystalline mineral of aluminum silicates and with potassium, sodium, calcium, or barium) and fired at high temperatures
Pottery	Clayware, esp. earthenware as distinguished on the one hand from porcelain and stoneware and on the other from brick and tile; objects made from fired clay
Stoneware	Strong, opaque ceramic ware that is high-fired, well vitrified, and nonporous; pottery or other objects made from fired clay
Tile	Flat or curved piece of fired clay
Vitrify	To convert into glass or a glassy substance by heat and fusion

**Table 2:** Identifiers for items with item descriptions and measurement information

Sample identifiers	Nuclide(s) identified	Description and measurement information (no further information was available for quoted marks)
Dishes		

Beatrix Potter		Cupped saucer, elevated, less than full interior contact, “The World of Beatrix Potter, Royal Albert Bone China, England, Beatrix Potter, c 1986, F. W. & Co.”
Blue Willow	^232^Th, ^238^U	Cupped saucer, less than full interior contact, “Japan”
English Garden	^232^Th, ^238^U	Rimmed saucer, elevated, less than full interior contact, “English Garden, Fine China, 1221, Japan”
Fiesta, green	^232^Th, ^235^U	Green oval tray, full contact on interior surface, “MPCo, 731C”
Fiesta, orange	^232^Th	Orange glue-joined cup/saucer, measured on bottom of domed saucer with unglazed rim smaller than ion chamber face dimensions
Haviland	^232^Th, ^238^U	Plate, full contact, interior surface, “Traditions Fine China, Made in Thailand for Johann Haviland China Corporation”
Haviland-Limoges		Plate, full contact on interior surface, “Haviland, France, Decorated by Haviland & Co., Limoges.” Measurements were made on top (/t) and underneath (/u).
Lenox	^232^Th, ^238^U	Rimmed saucer, elevated, less than full interior contact, “Lenox, Metropolitan Collection, Rose Manor, made in U.S.A.”
Monticello		Rimmed saucer, elevated, less than full interior contact, “Liberty Blue, Original Copper Engraving of Historic Colonial Scenes Printed on Staffordshire Ironstone, Detergent and Dishwasher Safe, Made in England, Monticello”
Noritake	^232^Th, ^238^U	Oval white bowl, full contact, interior surface, “Noritake, Japan, 6537, Royale Claret”
Pfaltzgraff	^232^Th, ^238^U	Rimmed saucer, elevated, less than full interior contact, “Pfaltzgraff, Copyright, U.S.A.”
Seaplate	^232^Th, ^238^U	Plate (smaller than contemporary size), full contact, interior surface, “J. W. Pankhurst & Co., Stone China”
Wedgewood, lg		Large rimmed plate, full contact, interior surface, “Wedgewood Bone China, Made In England, Cavendish, R4680”
Wedgewood, sm		Small rimmed plate, full contact, interior surface, “Royal Blue Ironstone, Enoch Wedgewood (Tunstall) Ltd., England, Trademark, Founded in 1838”
White bowl	^232^Th, ^238^U	Round white bowl, full contact on interior surface

Figures		

Angel	^232^Th, ^238^U	Small pottery angel figure, less than full contact on full-length, flowing robe, “Japan”
Bare wood		Construction plywood section
Church	^232^Th, ^238^U	Porcelain church figure, too small in any dimension for full contact, “Lenox”
Glaze, artist		Sample of beige paint from ceramics artist, painted on wood surface
Glaze, Mocha		Duncan Gallery Opaque Glaze, GO126, Mocha, “Safe for Food Containers”, painted on wood surface
Glaze, No Lead		Olevia Colors NO LEAD, 5025, “Food Safe”, painted on wood surface
Lid		Domed pottery greenware bowl lid with knob, less than full contact
Limoges	^232^Th, ^235^U	Sugar bowl, less than full contact on rectangular base with unglazed rim, “Limoges China Co., Sebring, Ohio, USA, Golden Glow, Pat. App. For”
Longaberger	^232^Th, ^238^U	Teapot, less than full contact on domed bottom with unglazed lip, “Longaberger Pottery, Made in the USA, Freezer/Microwave/Dishwasher/Oven Safe”
Poster		Shiny surface of foam poster board
Shard	^232^Th, ^238^U	Inside bottom of urn pottery shard, “China”

NBS Glass		

NBS glass large	^232^Th	Large square, 5 cm × 16 cm × 16 cm, orange colored, translucent
NBS glass medium	^232^Th	Medium rectangle, 5 cm 13 × cm × 18 cm, orange colored, translucent
NBS glass small		Small rectangle, 2 cm × 10 cm × 15 cm, greenish-orange colored, translucent, badly chipped

Tile and brick		

Tile, 1	^238^U	Tile #1 (17.8 cm), “Made in Mexico”
Tile, 2	^238^U	Tile #2 (15.2 cm), “FT, Made in USA”
Tile, 3	^238^U	Tile #3 (31.8 cm), “Made in Italy, Monocottura, 1200 ”
Tile, 4	^232^Th	Tile #4 (10.2 cm), “Made in USA”, measurements front and back (/b)
Tile, 5	^232^Th, ^238^U	Tile #5 (29.8 cm), “Made in Mexico, T12”, measurements front and back (/b)
Tile, foyer		NIST foyer brick, in place from original construction, unshielded and with paper shield (/p)
Tile, mosaic		Mosaic-style tile, handprinted “copy of mosaic at church site of the multiplication of loaves and fishes, Galilee, Israel, 1996”
Tile, NIST new		Current NIST construction tile, stock sample, unshielded and with paper shield (/p)
Tile, rough		Rough tile (22.2 cm), (indecipherable characters) “Made in Italy”, measurements front and back (/b)
Tile, stairwell		NIST stairwell tile, in place from original construction, unshielded and with paper shield (/p)

**Table 3a:** Concentrations and standard uncertainties, i.e., standard deviations, for samples with properties amenable to full analysis

Sample	EIC data		Ratio EIC/ZnS	ZnS data	
	Net concentration (Bq/g)	Uncertainty (Bq/g)		Net concentration (Bq/g)	Uncertainty (Bq/g)
Tile, NIST new /p	0.011	0.001	≫1.0	0.000	0.000
Bare wood	0.025	0.033	1.8	0.014	0.016
Haviland/Limoges /u	0.037	0.009	1.1	0.033	0.008
Poster	0.038	0.004	0.8	0.047	0.015
Haviland/Limoges /t	0.043	0.011	1.6	0.026	0.007
NBS glass small	0.055	0.007	1.0	0.055	0.015
Noritake	0.061	0.001	1.5	0.040	0.017
Lenox	0.063	0.019	0.8	0.078	0.090
Sea plate	0.076	0.021	1.4	0.052	0.017
Glaze, artist	0.079	0.003	1.2	0.068	0.017
Tile, #4 /b	0.083	0.002	0.9	0.089	0.015
Tile, mosaic	0.084	0.002	2.3	0.037	0.015
White bowl	0.110	0.026	1.3	0.086	0.026
Haviland	0.130	0.003	2.4	0.053	0.017
Tile, rough /b	0.136	0.003	0.8	0.166	0.046
Wedgewood, sm	0.147	0.003	2.8	0.052	0.016
English Garden	0.147	0.003	1.6	0.094	0.019
Tile, #5 /b	0.148	0.006	1.6	0.094	0.017
Monticello	0.151	0.003	4.5	0.034	0.015
Wedgewood, lg	0.162	0.003	1.4	0.117	0.018
Shard	0.181	0.025	1.6	0.114	0.016
Tile, #5	0.388	0.027	1.4	0.278	0.016
Pfaltzgraff	0.458	0.062	1.1	0.409	0.060
Tile, #2	0.551	0.040	1.1	0.485	0.067
Tile, rough	0.596	0.012	1.3	0.477	0.037
Tile, NIST new	0.625	0.044	1.3	0.491	0.016
Tile, #1	0.648	0.020	1.1	0.583	0.032
Tile, #4	0.650	0.041	1.2	0.529	0.081
Glaze, NoLead	0.691	0.071	1.2	0.597	0.020
Tile, #3	0.806	0.048	1.0	0.821	0.020
Glaze, Mocha	1.246	0.048	1.1	1.102	0.036
Fiesta, green	13.564	0.709	1.7	8.160	4.980
NBS glass large	103.267	9.981	0.8	122.213	1.325
NBS glass medium	376.118	20.014	0.9	418.812	2.453

**Table 3b:** Concentrations and standard uncertainties, i.e., standard deviations, for samples with vertical surfaces that preclude use of the ZnS system

Sample	EIC data	
	Net concentration (Bq/g)	Uncertainty (Bq/g)
Tile, stairwell/p	0.047	0.001
Tile, foyer/p	0.123	0.003
Tile, stairwell	0.604	0.013
Tile, foyer	0.888	0.019

**Table 3c:** Concentrations for samples with geometries that preclude uncertainty determinations

Sample	EIC data Net concentration (Bq/g)	Ratio EIC/ZnS	ZnS data Net concentration (Bq/g)
Beatrix Potter	0.083	7.5	0.011
Blue Willow	0.142	1.0	0.141
Church	0.216	1.7	0.127
Lid	0.251	1.4	0.180
Longaberger	0.586	1.3	0.459
Angel	0.996	5.5	0.179
Limoges	37.051	2.2	16.562
Fiesta, orange	204.490	1.7	122.332

## Footnotes

1 Certain commercial equipment, instruments, or materials are identified in this paper to foster understanding. Such identification does not imply recommendation or endorsement by the National Institute of Standards and Technology, nor does it imply that the materials or equipment identified are necessarily the best available for the purpose.

2 Canberra Industries Model Genie 2000.

3 Ludlum Measurements Model 2200 Scaler/Ratemeter with a Model 43-1 probe, with the gridded probe face masked to provide a circular open area of 19.6 cm^2^.

4 Rad Elec, Inc. Model “S” Eperm chamber with “ST” electret, chamber separated at midline to form a cylindrical ion chamber.

## References

[1] Jensen D (1952). Uranium glass and its fluorescence. Rocks and Minerals.

[2] (1987). NCRP Report No. 95, Radiation exposure of the US population from consumer products and miscellaneous sources.

[3] (1993). Code of Federal Regulations, Certain items containing byproduct material, 10 CFR 30.15.

[4] (1970). Code of Federal Regulations, Unimportant quantities of source material, 10 CFR 40.13.

[5] Searle AB (1921). The Clayworker’s Hand-Book.

[6] Singer F, Singer SS (1963). Industrial Ceramics.

[7] Rhodes D (1972). Clay and Glazes for the Potter.

[8] Chappell J (1977). The Complete Book of Clay and Glazes.

[9] Youden WJ (1994). Experimentation and measurement.

[10] Natrella MG (1966). Experimental statistics.

[11] Currie LA (1968). Limits for qualitative detection and quantitative determination. Anal Chem.

[b12-j52hob] 12K. Eberhardt, private communication.

[b13-j52hob] 13L. A. Currie, private communication.

